# Relationship of the balloon analog risk task to neurocognitive impairment differs by HIV serostatus and history of major depressive disorder

**DOI:** 10.1007/s13365-021-01046-z

**Published:** 2022-01-03

**Authors:** Rowan Saloner, Erin E. Morgan, Mariam A. Hussain, David J. Moore, Robert K. Heaton, Mariana Cherner, Igor Grant, Jennifer E. Iudicello, the TMARC Group

**Affiliations:** 1 Joint Doctoral Program in Clinical Psychology, San Diego State University/University of California, San Diego, San Diego, CA USA; 2 Department of Psychiatry, HIV Neurobehavioral Research Program, University of California, San Diego, San Diego, CA USA; 3 Department of Neurology, Memory and Aging Center, University of California, San Francisco, San Francisco, CA USA

**Keywords:** HIV-associated neurocognitive disorders, Depression, Risk-taking, Cognition, HIV risk, Decision making

## Abstract

HIV and major depressive disorder (MDD) commonly co-occur and are both linked to greater risk-taking behavior, possibly due to neurocognitive impairment (NCI). The present study examined the concordance of the Balloon Analog Risk Task (BART), a gold standard measure of risk-taking propensity, with NCI and real-world sexual risk behaviors in PWH with comorbid MDD. Participants included 259 adults, stratified by HIV serostatus (HIV + /HIV −) and lifetime MDD (MDD + /MDD −), who completed neuropsychological testing, the BART, and sexual risk behavior questionnaires. Logistic regression, stratified by HIV serostatus, examined joint effects of MDD and BART (linear and quadratic) on NCI. Follow-up linear regressions examined sexual risk behavior and neurocognitive domain T-scores as correlates of the BART. NCI prevalence was lowest in HIV − /MDD − , but BART scores did not differ by HIV/MDD status. In the HIV + group, BART performance predicted NCI such that high and low BART scores related to greater odds of NCI, but only in dual-risk HIV + /MDD + individuals. HIV + /MDD + individuals with both low and high BART scores exhibited poorer learning and recall, whereas processing speed and executive function were only poor in low BART risk-taking HIV + /MDD + . Higher BART scores linearly related to higher sexual risk behaviors only in MDD + individuals, independent of HIV serostatus. Low and high risk-taking on the BART may reflect discrete neurocognitive profiles in HIV + /MDD + individuals, with differential implications for real-world sexual risk behavior. HIV and comorbid MDD may disturb corticostriatal circuits responsible for integrating affective and neurocognitive components of decision-making, thereby contributing to risk-averse and risk-taking phenotypes.

## Introduction

HIV disease is no longer considered a terminal illness due to effective antiretroviral therapy (ART). Accordingly, the clinical care for persons with HIV (PWH) has now shifted toward adherence to ART, treatment of HIV-related comorbidities, and monitoring of behaviors that increase risk for HIV transmission. Early in the course of the disease, HIV is capable of infiltrating the central nervous system (CNS) and producing neuroimmunological insults, particularly in the presence of ART regimens that have poor CNS penetration (Ellis et al. 2007; Hult et al. 2008). Although the neuroinflammatory cascades that drive HIV neuropathologenesis can diffusely impact the CNS, frontostriatal circuits that support higher-order neurocognitive functions, emotional regulation, and reward processing are particularly vulnerable to HIV-related neural injury (Soontornniyomkij et al. 2016; Woods et al. 2009). Roughly 30–50% of PWH present with neurocognitive impairment (NCI; Heaton et al. 2010; Saloner and Cysique 2017). In addition to premorbid psychosocial risk factors that precede the acquisition of HIV, acquired frontostriatal injury due to HIV is thought to contribute to the high prevalence of NCI and neuropsychiatric disturbances, including major depressive disorder (MDD) and substance use disorders (Anand et al. 2010; Arseniou et al. 2014; Ipser et al. 2015). An estimated 22–54% of PWH meet clinical criteria for a lifetime diagnosis of MDD (Rabkin 2008; Rooney et al. 2019; Rubin and Maki 2019), which can impact real-world outcomes such as ART adherence and employment (Heaton et al. 2004a; Rabkin 2008).

Group level comparisons on standard neuropsychological tests between PWH and HIV-seronegative (HIV −) counterparts often demonstrate HIV-associated deficits in episodic memory (typically mixed encoding and retrieval profile), cognitive and psychomotor slowing, poor complex attention/working memory, and executive dysfunction (including novel problem solving and set-shifting; Woods et al. 2009). Recent studies have also begun to incorporate experimental cognitive neuropsychology paradigms into neurobehavioral assessment protocols to tap into multi-faceted constructs that more closely resemble real-world situations than traditional clinical neuropsychological measures (Llewellyn 2008; Woods et al. 2009). Risk-taking is one such construct that has received considerable attention, given that the likelihood of acquiring or transmitting HIV is heightened by the decision to engage in risky behaviors such as unprotected sex and injection drug use (Crooks et al. 2015; Iudicello et al. 2013; Montoya et al. 2016).

The Iowa Gambling Task (IGT; Bechara 2007; Bechara et al. 1994) is the most commonly studied experimental measure of risky decision-making in PWH. The IGT requires participants to choose from one of four card decks, with two disadvantageous “risky” decks reflecting larger, immediate rewards but higher long-term penalties and two advantageous “safe” decks reflecting smaller, immediate rewards but higher long-term earnings. Studies examining the neuropsychological correlates of IGT performance in PWH with and without comorbid substance use diagnoses demonstrate “riskier” decision-making in PWH, particularly among individuals with deficits in inhibitory control, learning, and memory (Hardy et al. 2006; Iudicello et al. 2013; Martin et al. 2013; Martin et al. 2004). Notably, several studies have demonstrated that the concordance of the IGT with standard neuropsychological measures and real-world risk behaviors is influenced by affective factors. Thames and colleagues demonstrated a mediating effect of depression on the relationship between executive function and IGT performance in PWH (Thames et al. 2012), while several studies have identified moderating effects of emotional distress and sensation seeking personality traits on the relationship between IGT performance and sexual risk behaviors and substance use in PWH (Golub et al. 2016; Gonzalez et al. 2005; Wardle et al. 2010).

The Balloon Analog Risk Task (BART) is another common laboratory-based assessment of risk-taking that requires participants to make sequential decisions about whether to progressively pump up a balloon to earn money or stop pumping and collect their earnings (Lejuez et al. 2002). Each sequential pump results in a fixed and known monetary gain but also an increase in the unknown probability that the balloon explodes, which would result in the loss of previously accrued monetary gains. The BART is less dependent upon an individual’s ability to learn reward and punishment contingencies than the IGT and is therefore considered a more direct measure of risk-taking propensity (Hevey et al. 2017; Llewellyn 2008), which is defined as the tendency to engage in behaviors that may yield positive outcomes but also carry an uncertain likelihood of negative outcomes (Balogh et al. 2013; Kreek et al. 2005). Participants do not have to learn which behaviors are risky – they are explicitly told each pump will result in a fixed gain or balloon pop and therefore the BART is more reflective of real world risk-taking in which individuals willingly engage in behaviors that expose themselves to potential punishment in the pursuit of rewards (Bishara et al. 2009). The average number of pumps on unexploded balloons is the primary score used to measure BART performance (i.e., risk-taking propensity), and this metric has been found to converge with real-world risk behaviors (e.g., substance use, unprotected sex; Hunt et al. 2005; Lejuez et al. 2002, 2004).

Both low and high pumps may indicate suboptimal BART performance, but due to different mechanisms. Several studies have reported significantly higher BART pumping tendencies in PWH compared to HIV- controls, which has been interpreted as a greater propensity toward risk-taking (Meade et al. 2016; Paydary et al. 2016). In these studies, poorer performance (i.e., higher average number of pumps) was linked to higher levels of impulsivity (Paydary et al. 2016) and altered functioning in brain regions affected by HIV and linked to decision-making (e.g., prefrontal cortex and anterior cingulate; Meade et al. 2016). However, studies in populations with acquired brain injuries, including those in which risk-taking and poor decision-making are clinical hallmarks (e.g., frontotemporal dementia), frequently observe *fewer* pumps in patients compared to healthy controls (Balagueró et al. 2016; Fecteau et al. 2013; Strenziok et al. 2011). Although lower BART pumps reduces the likelihood of a balloon explosion, these observations in patients with NCI are interpreted as impaired stimulus-reinforcement learning, given that lower risk-taking will also reduce potential earnings. Research from computational cognitive models also suggests that lower BART pumps may manifest from greater perceived probability of losing (loss sensitivity) and less consistent decision-making (Bishara et al. 2009; Kahneman and Tversky 1979).

Despite being considered a gold-standard measure of risk-taking propensity, little is known about the concordance of the BART with standard neuropsychological performance and HIV transmission risk behaviors in PWH, as well as the moderating role of depression on these relationships. Moreover, the majority of studies have assumed linear associations between the BART and other aspects of neurobehavior, despite evidence that risk aversion and risk-taking can both be indicative of neurocognitive and affective dysfunction (James et al. 2015; Smoski et al. 2008; Whittle et al. 2015). The present study leveraged comprehensive neurobehavioral data from a cohort study of PWH and HIV- individuals to evaluate linear and non-linear relationships of the BART with NCI and HIV transmission risk behaviors. Moreover, we examined whether these relationships were moderated by depression, defined by clinical diagnoses of lifetime MDD. Consistent with the existing literature, we hypothesized that both low and high levels of risk-taking propensity (i.e., BART performance) would relate to higher odds of NCI compared to intermediate levels of risk-taking propensity, and that higher levels of risk-taking propensity would be associated with increased HIV transmission risk behaviors. In addition, we hypothesized that the relationships between risk-taking propensity, NCI, and HIV transmission risk behaviors would be strongest in PWH with MDD reflecting greater disturbance in the cognitive and affective components of risk taking and decision-making.

## Materials and methods

### Participants

Participants were 131 HIV-seropositive (HIV +) and 128 HIV − adults enrolled in the University of California San Diego’s (UCSD) Translational Methamphetamine AIDS Research Center (TMARC), a NIDA-funded cohort study focusing on the effects of HIV and methamphetamine (METH) on neurobehavioral functioning. Given the present study’s focus on the relationship between risk-taking and neurocognition in the context of HIV and depression, METH use disorder (defined by TMARC as a history of METH dependence with abuse or dependence within the past 18 months, as diagnosed by the Composite International Diagnostic Interview [CIDI] (World Health Organization 1998)) was considered as a covariate while participants were stratified by HIV and lifetime MDD diagnoses into four groups: HIV − /MDD − (*n* = 92), HIV − /MDD + (*n* = 36), HIV + /MDD − (*n* = 75), and HIV + /MDD + (*n* = 56). Participants provided written informed consent to study procedures, which were approved by the UCSD Institutional Review Board. Potential participants were excluded if they reported a history of a psychotic or mood disorder with psychotic features, or had a neurological (e.g., stroke, seizure disorder) or non-HIV medical condition (e.g., hepatitis C) that may confound neurobehavioral test results. Given the overarching cohort study aims, TMARC criteria excluded participants with recent alcohol dependence (within the last 12 months) or recent diagnoses of abuse (within the last 12 months) or dependence (within the last 5 years) for all other substances except METH and cannabis.

### Neuromedical assessment

All participants underwent a comprehensive neuromedical assessment, blood draw, and lumbar puncture. HIV disease was diagnosed by enzyme-linked immunosorbent assay (ELISA) with Western blot confirmation. Among PWH, plasma HIV RNA was measured using reverse transcriptase-polymerase chain reaction (Amplicor, Roche Diagnostics, Indianapolis, IN) and deemed undetectable at a lower limit of quantitation (LLQ) of 50 copies/ml.

### Neuropsychiatric assessment

To determine if participants had experienced a clinically significant history of depressed mood, the CIDI (World Health Organization 1998) was administered by trained psychometrists to establish DSM-IV lifetime and current (within the last 30 days) diagnoses of MDD. For the present analysis, participants who met criteria for a lifetime MDD diagnosis were grouped as MDD + , given that few participants met criteria for current MDD (*n* = 15). The CIDI was also used to diagnose lifetime and current substance use disorders and Antisocial Personality Disorder.

Participants also completed several self-report measures to characterize recent levels of depressive symptomatology in the MDD + and MDD − groups. Specifically, the total score from the second edition of the Beck Depression Inventory-II (BDI-II; Beck et al. 1996) was used to measure the severity of overall symptoms of depression over the 2 weeks prior to the study visit. Consistent with the methodology reported by Marquine et al. (2014), a composite apathy score was also generated by combining apathy-related items from the BDI-II (i.e., loss of pleasure, loss of interest, difficulty making decisions, and feelings of tiredness and fatigue), the “after illness” apathy subscale from the Frontal Systems Behavior Scale (FrSBe; Grace, 2001), and the vigor-activity subscale from the Profile of Mood States (McNair 1992). Raw scores on the individual subscales were converted to z-scores based on the mean and standard deviation of the entire TMARC study healthy control group (i.e., HIV-/METH-), and z-scores were then averaged and converted to T-scores. Higher apathy T-scores represent higher levels of apathy.

Using the same composite approach, T-scores were also derived for non-depressive “frontal systems” traits of impulsivity/disinhibition and sensation-seeking (Marquine et al. 2014), which have been shown to influence risky decision-making in PWH (Gonzalez et al. 2005; Paydary et al. 2016; Wardle et al. 2010). Impulsivity/disinhibition T-scores were derived from the ‘after illness’ disinhibition subscale from the FrSBe, the urgency and lack of premeditation subscales from the UPPS Impulsive Behavior Scale (Whiteside and Lynam 2001), and the Barratt Impulsiveness Scale total score (Patton et al. 1995). Sensation-seeking T-scores were derived from the Kalichman sexual and non-sexual sensation-seeking scales (Kalichman et al. 1994; Kalichman and Rompa 1995). Higher T-scores represent greater impulsivity/disinhibition or sensation-seeking traits.

### Neurobehavioral assessment

#### Neuropsychological testing

All participants completed a comprehensive and standardized neuropsychological assessment including an estimate of premorbid verbal IQ (i.e., Wide Range Achievement Test Reading subtest, Version 4 (Wilkinson and Robertson 2006) and seven neurocognitive domains commonly impacted by HIV (Heaton et al. 2010; Morgan et al. 2012; Rippeth et al. 2004). The domains and individual tests were: *verbal fluency* (Controlled Oral Word Association Test, animal fluency, action fluency), *processing speed* (Trail Making Test A, WAIS-III Digit Symbol, WAIS-III Symbol Search, Stroop Color and Word Test Color Score), *executive function* (Wisconsin Card Sorting Test-64 Card Version, Trail Making Test B, Stroop Color and Word Test Interference Score), *learning* and *delayed recall* (Hopkins Verbal Learning Test-Revised, Brief Visuospatial Memory Test-Revised), *working memory* (WMS-III Spatial Span, Paced Auditory Serial Addition Test), and *complex motor skills* (Grooved Pegboard Test). For participants who had been exposed to the test battery at prior research visits, raw scores for each test were converted to practice effect-adjusted scaled scores (Cysique et al. 2011). Using the most comprehensive normative standards available, scaled scores were converted to demographically-corrected T-scores that adjusted for the effects of age, education, sex, and race/ethnicity, as appropriate (Heaton et al. 2004b, 2003; Norman et al. 2011). Individual test T-scores were averaged within each domain to derive domain-specific T-scores, which were examined as secondary outcomes in the present study. In order to classify neurocognitive impairment, the primary outcome of interest, T-scores were converted to deficit scores that give differential weight to impaired, as opposed to normal scores, on a scale ranging from 0 (*T* ≥ 40; normal) to 5 (*T* < 20; severe impairment). Deficit scores were averaged across the entire test battery to derive a global deficit score (GDS). Consistent with prior studies, the presence of global neurocognitive impairment was classified as GDS ≥ 0.5. (Blackstone et al. 2012; Carey et al. 2004).

#### Balloon analog risk task

The BART is a computer-simulated measure of risky decision making that has strong convergent validity with real-world risk behaviors and self-report trait-measures of risk-taking tendencies (Hunt et al. 2005; Lejuez et al. 2002). The present study employed the same BART paradigm as described in detail by Hunt et al. (2005). Briefly, participants have the opportunity to earn simulated money by clicking a balloon pump button that inflates a simulated balloon presented on the computer screen. Participants earn 1 cent of simulated money per balloon pump. However, they are informed that the balloon will explode at some point, that the exact explosion point will vary across trials, and that they will lose money accrued on a trial if the balloon explodes. Thus, each trial ends when 1) the participant decides to stop pumping and collect money or 2) the balloon explodes. The maximum number of possible pumps for any given trial is 128 and the probability of explosion, *x*, on a given pump, *i*, is *x* = 1/[(128 − *i*) + 1)]. Thus, each additional pump is associated with a higher likelihood of losing financial gains as well as a decrease in the relative gain to be earned (i.e., diminished rewards). Participants completed 30 trials and the explosion point on each specific trial was the same across all participants. At the end of the study visit, participants were awarded actual money (up to $15) that was proportional to the simulated money they earned on the BART. The average number of balloon pumps on trials in which the balloon did not explode was used as the primary predictor for analyses (i.e., “BART adjusted pumps”). The adjusted pumps value is the most commonly used index of risk-taking propensity on the BART because it is not confounded by the fact that explosion points vary across balloon trials (Lauriola et al. 2014). The total number of trials with explosions is also provided as an additional metric for descriptive purposes.

### HIV transmission risk behavior T-scores

In order to capture real-world risky decision-making that holds clinical relevance, participants completed questionnaires assessing for risky sexual behaviors: (1) Sexual Risk Scale subscore from the Modified Risk Assessment Battery (RAB; Navaline et al. 1994), and (2) Sexual Risk Scale total score (SRS; DeHart and Birkimer 1997). On the Modified RAB Sexual Risk Scale, participants rate engagement in sexual risk behaviors (e.g., frequency of unprotected sex) over the past 6 months, with higher scores representing greater sexual risk-taking. The SRS measures attitudes, norms, intention, and expectations related to practicing safer sex, with higher total scores suggesting a stronger intention to practice safe sex. For ease of interpretation, the total SRS score was reverse-coded to align with the RAB such that higher scores represented riskier intentions. Employing the same methodology as the “frontal systems” behaviors T-scores approach described above (Marquine et al. 2014), RAB and SRS scores were converted to z-scores based on the entire TMARC study control group and then averaged to form a composite HIV transmission risk behaviors T-score. Higher T-scores represent a greater propensity to engage in risky sexual behaviors that enhance risk for HIV acquisition and transmission. HIV transmission risk behaviors T-scores were used as a secondary outcome in analyses examining the ecological validity of the BART risk-taking index.

### Statistical analysis

HIV/MDD group comparisons on demographic, psychiatric, substance use, HIV disease characteristics (HIV + stratum only), and neurobehavioral variables were performed with ANOVA, Wilcoxon rank-sum tests, or likelihood ratio *χ*^2^ tests, as appropriate. Stepwise multivariable logistic regression models that examined the interactive effects of MDD and risk-taking (i.e., BART adjusted pumps) on odds of NCI were conducted separately for each HIV stratum. Variables that demonstrated univariable trend-level associations (*p* < 0.10) with the independent variables (MDD group or BART adjusted pumps) or the primary dependent variable (NCI) were entered as covariates in step 1. Depression and apathy scales (i.e., BDI-II score and apathy T-scores) were reported to further characterize the clinical MDD groups, but were not considered as candidate covariates given the high degree of overlap with the construct of depression captured by the clinical MDD groups. The optimal combination of covariate predictors in step 1 were determined based on Akaike information criteria (AIC) with backwards selection (Akaike, 1974; Burnham and Anderson 2004). The main effect of MDD group and the linear and quadratic effects of BART adjusted pumps were entered in step 2. If non-significant (*p* < 0.05), the quadratic adjusted pumps term was removed in step 2 and the model was re-run to obtain an appropriate estimate of the linear adjusted pumps term. Last, the interaction(s) between MDD group and the BART adjusted pumps term(s) were entered in step 3. This stepwise approach allowed us to determine the incremental contributions of the independent main effects and interactions of interest above and beyond the contributions of relevant covariates. These stepwise regression analyses were stratified by HIV serostatus given the limited power to detect a 4-way interaction effect (i.e., HIV × MDD × [adjusted pumps × adjusted pumps]). Nagelkerke pseudo-*R*^2^ goodness-of-fit statistics for a categorical dependent variable (i.e., NCI) are reported to facilitate comparisons between the nested stepwise logistic regression models (Nagelkerke 1991). Odds ratios (OR) are reported as effect size estimates for individual parameters in logistic regression analyses.

To determine which neurocognitive domains were driving any significant interactions of MDD and BART adjusted pumps on the global NCI classification, follow-up linear regression analyses examined the seven neurocognitive domain-specific T-scores as separate outcomes. In order to limit the Type I error rate for these seven additional analyses, we used the false discovery rate (FDR) method and set the FDR at 5% (Benjamini and Hochberg 1995). Last, to determine the ecological validity of the laboratory-based BART index of risk-taking propensity for clinically-relevant real-world risk behaviors, we examined relationships between BART adjusted pumps and HIV transmission risk behavior T-scores across the entire study sample and within the four study groups. All analyses were conducted using JMP Pro version 14.0.0 (JMP^®^, Version < 12.0.1 > . SAS Institute Inc., Cary, NC, 2018).

## Results

### Study group characteristics

The full study sample was 56% non-Hispanic White and 81% male with a mean age of 41.8 years and mean education of 13.8 years. Participant characteristics by MDD group, stratified by HIV serostatus, are presented in Table 1. Age, sex, years of education, estimated premorbid verbal IQ, and race/ethnicity were comparable between MDD- and MDD + groups in both HIV strata (*p*s > 0.069). In the HIV − stratum, MDD + participants exhibited higher rates of METH use disorders (OR = 5.12, *p* < 0.001) and lifetime non-METH use disorders (OR = 3.33, *p* = 0.011) than MDD- participants; substance use characteristics did not significantly differ by MDD group in the HIV + stratum (*p*s > 0.140). Similarly, HIV disease characteristics did not differ by MDD group in the HIV + stratum (*p*s > 0.240), with the majority of participants on ART medication (81%) and virally suppressed (70%).

**Table 1:** Study group characteristics

Variable	HIV −			HIV +		
	MDD − (*n* = 92)	MDD + (*n* = 36)	*p*	MDD − (*n* = 75)	MDD + (*n* = 56)	*p*
Demographics						
Age (years)	41.1 (14.5)	39.6 (13.6)	.573	42.3 (13.4)	43.9 (11.1)	.467
Sex (male)	63 (68.5%)	23 (63.9%)	.773	71 (94.7%)	52 (92.9%)	.953
Education (years)	13.7 (2.5)	12.8 (2.5)	.070	14.2 (2.5)	14.1 (2.3)	.844
Estimated premorbid verbal IQ	102.2 (13.0)	100.2 (14.5)	.451	102.0 (13.2)	104.3 (10.3)	.284
Race/ethnicity			.752			.142
Non-Hispanic White	50 (54.3%)	21 (58.3%)		38 (50.7%)	36 (64.3%)	
Black	14 (15.2%)	6 (16.7%)		12 (16.0%)	7 (12.5%)	
Hispanic	21 (22.8%)	6 (16.7%)		23 (30.7%)	9 (16.1%)	
Asian	2 (2.2%)	2 (5.6%)		0 (0.0%)	2 (3.6%)	
Other	5 (5.4%)	1 (2.8%)		2 (2.7%)	2 (3.6%)	
Depression characteristics						
Current major depressive disorder	0 (0.0%)	4 (11.1%)	.007	0 (0.0%)	11 (19.6%)	< .001
BDI-II score	3 [0, 13]	6 [3, 20]	.021	7 [2, 15]	15 [5, 26]	.001
Apathy T	56.4 (14.7)	62.3 (16.7)	.052	63.9 (19.3)	72.3 (18.7)	.018
Frontal systems behaviors						
Impulsivity/disinhibition T	56.8 (15.0)	60.7 (14.7)	.186	59.2 (14.2)	59.8 (11.8)	.802
Sensation-seeking behaviors T	51.9 (11.0)	49.2 (8.0)	.189	53.8 (9.8)	52.7 (9.6)	.518
ASPD	16 (17.6%)	11 (30.6%)	.116	9 (12.0%)	10 (17.9%)	.349
Alcohol and substance use						
METH use disorder	31 (33.7%)	26 (72.2%)	< .001	33 (44.0%)	26 (46.4%)	.921
Lifetime non-METH SUD	12 (13.0%)	12 (33.3%)	.011	10 (13.3%)	5 (8.9%)	.428
Lifetime alcohol use disorder	39 (42.4%)	21 (58.3%)	.104	35 (46.7%)	19 (33.9%)	.141
Tobacco smoking history			.292			.623
Current	20 (21.7%)	7 (19.4%)		16 (21.3%)	16 (28.6%)	
Past	43 (46.7%)	22 (61.1%)		38 (50.7%)	25 (44.6%)	
Never	29 (31.5%)	7 (19.4%)		21 (28.0%)	15 (26.8%)	
HIV disease characteristics						
AIDS diagnosis				33 (44.0%)	25 (44.6%)	.942
Duration of HIV infection (years)				6.7 [1.6, 15.2]	7.5 [2.4, 17.4]	.550
Nadir CD4 count (cells/mm^3^)				250 [107, 373]	300 [150, 443]	.241
Current CD4 count (cells/mm^3^)				591 [344, 783]	565 [467, 793]	.289
Detectable plasma viral load				19 (26.4%)	20 (35.7%)	.257
On ART				62 (82.7%)	44 (78.6%)	.556

Values are presented as mean (SD), median [IQR], or *n* (%)

*ART* antiretroviral therapy, *ASPD* antisocial personality disorder, *BDI-II* Beck Depression Inventory version two, *SUD* substance use disorder

MDD + participants expectedly had higher (but still overall low) rates of current MDD (HIV − : 11%, HIV + : 20%) and higher BDI-II (Cohen’s *d* = 0.55, *p* < 0.001) and apathy scores (*d* = 0.48, *p* < 0.001) than MDD − participants, regardless of HIV serostatus. Overall scores on the scales measuring recent depressive symptoms were significantly higher in HIV + participants compared to HIV − participants (BDI-II: *d* = 0.39, *p* = 0.002; apathy: *d* = 0.53, *p* < 0.001). Although the HIV + /MDD − group by definition had never met criteria for a lifetime (or current) MDD diagnosis, this group had similar BDI-II and apathy scores compared to the HIV − /MDD + group (*p*s > 0.480).

### Neurobehavioral performance by HIV/MDD group

Table 2 reports performance on neuropsychological testing, the BART, and HIV transmission risk behaviors across HIV/MDD groups. The prevalence of global NCI was 31% in the full study sample. A one-way likelihood ratio *χ*^2^ test indicated a trend-level omnibus difference in the prevalence of global NCI across the four groups (*χ*^2^ = 6.90, *p* = 0.075). Compared to HIV − /MDD − (21%), the odds of NCI were roughly twice as high in the three risk groups: HIV − /MDD + (36%, OR = 2.17, *p* = 0.073), HIV + /MDD − (35%, OR = 2.04, *p* = 0.044), HIV + /MDD + (38%, OR = 2.31, *p* = 0.027). Rates of NCI did not differ between the three risk groups (OR range: 0.94 to 1.13). A similar pattern was generally observed for domain-specific performance (refer to Table 2). Significant omnibus HIV/MDD effects were detected for processing speed (*F* = 3.45, *p* = 0.017, *η*^*2*^ = 0.039) and executive functioning T-scores (*F* = 4.30, *p* = 0.006, *η*^*2*^ = 0.048), with HIV − /MDD − participants exhibiting higher processing speed T-scores compared to HIV + /MDD − (*d* = 0.45, *p* = 0.005) and HIV + /MDD + (*d* = 0.42, *p* = 0.013) as well as higher executive functioning T-scores compared to all three risk groups (vs. HIV − /MDD + : *d* = 0.45, *p* = 0.022; vs. HIV + /MDD − : *d* = 0.43, *p* = 0.007; vs. HIV + /MDD + : *d* = 0.51, *p* = 0.003).

**Table 2:** Neurocognitive performance and risk-taking by HIV and MDD group

Variable	HIV − /MDD − (*n* = 92)	HIV − /MDD + (*n* = 36)	HIV + /MDD − (*n* = 75)	HIV + /MDD + (*n* = 56)	*p*
Neuropsychological testing					
Global neurocognitive impairment	19 (20.7%)	13 (36.1%)	26 (34.7%)	21 (37.5%)	.075
Verbal fluency T	50.2 (8.3)	50.6 (8.6)	47.2 (8.1)	49.2 (7.9)	.079
Processing speed T^a^	51.6 (8.5)	49.3 (8.4)	48.0 (7.0)	48.2 (8.0)	.017
Executive functioning T^b^	51.2 (8.9)	47.3 (9.2)	47.5 (8.1)	46.8 (8.7)	.006
Learning T	44.5 (8.0)	43.8 (11.0)	42.0 (7.9)	42.2 (8.3)	.188
Delayed recall T	45.1 (8.8)	44.9 (9.7)	43.5 (8.8)	43.8 (8.3)	.642
Working memory T	49.7 (8.3)	47.4 (8.8)	46.7 (9.1)	46.4 (7.6)	.066
Motor T	50.5 (9.9)	49.9 (10.4)	49.3 (10.0)	48.1 (9.6)	.538
Risk taking					
Balloon Analog Risk Task					
Adjusted average pumps	28.7 (14.9)	29.3 (12.9)	29.9 (14.8)	30.9 (12.9)	.826
Total explosions	6 [3, 10]	6 [4, 9]	6 [3, 9]	7 [4, 10]	.853
HIV transmission risk behaviors T^c^	51.8 (11.7)	53.6 (12.9)	64.8 (14.5)	64.2 (12.8)	< .001

Values are presented as mean (SD), median [IQR], or *n* (%). *p* values represent omnibus HIV/MDD group effects on neurobehavioral outcomes. For significant omnibus effects (*p* < .05), pairwise comparisons were conducted and reported differences are significant at *p* < .05

^a^Pairwise comparisons indicated significantly higher processing speed T-scores in HIV − /MDD − compared to HIV + /MDD − and HIV + /MDD +

^b^Pairwise comparisons indicated significantly higher executive functioning T-scores in HIV − /MDD − compared to HIV − /MDD + , HIV + /MDD − , and HIV + /MDD +

^c^Pairwise comparisons indicated significantly higher HIV transmission risk behavior T-scores in HIV + /MDD − and HIV + /MDD + compared to HIV − /MDD − and HIV − /MDD +

In the full study sample, the average number of BART pumps on trials without explosions (i.e., adjusted pumps) was 29.6 (SD = 14.1) and the median number of explosions was 6 (IQR: 4 to 10). HIV/MDD groups did not significantly differ with respect to average/median adjusted pumps (*p* = 0.826) or total explosions (*p* = 0.853). On the HIV transmission risk behaviors composite T-score, HIV + participants reported higher HIV transmission risk behaviors than HIV − participants (Cohen’s *d* = 0.95, *p* < 0.001), regardless of MDD status. MDD groups, however, did not univariably differ on HIV transmission risk behaviors within either HIV stratum (*p*s > 0.449).

### Interactive effects of MDD and BART on NCI

Across the full study sample, univariable logistic regression analysis indicated a significant linear association between BART adjusted pumps and lower odds of NCI (for 1 SD-unit increase: OR = 0.64, *p* = 0.002). Stepwise, multivariable logistic regression models stratified by HIV serostatus examined whether MDD moderated the relationship between BART adjusted pumps and odds of NCI. Table 3 presents the results of these stepwise models. In the HIV- stratum, the overall AIC-guided step 1 model was significant (*χ*^2^ = 10.44, Nagelkerke pseudo-*R*^2^ = 0.12, *p* = 0.005). With respect to retained covariates, higher sensation seeking behavior T-scores related to lower odds of NCI and a lifetime alcohol use disorder diagnosis related to higher odds of NCI. Step 2 entered MDD status and linear and quadratic adjusted pumps terms as predictors of NCI. After removing the non-significant quadratic adjusted pumps term (*p* = 0.812), the overall model fit of step 2 was marginally improved from step 1 (*χ*^2^ = 15.72, Nagelkerke pseudo-*R*^2^ = 0.17, *p* = 0.003, log-likelihood change *p* = 0.071) and indicated that neither MDD nor the linear effect of adjusted pumps significantly contributed to the probability of NCI above and beyond the covariates retained in step 1. Similarly, the interaction between MDD and the linear effect of adjusted pumps was not significant (*p* = 0.758) in step 3 and did not improve overall model fit (*χ*^2^ = 15.77, Nagelkerke pseudo-*R*^2^ = 0.17, *p* = 0.008, log-likelihood change *p* = 0.757).

**Table 3:** Step-wise logistic regression models examining the interactive effects of MDD and BART pumps on odds of NCI by HIV serostatus

Group: HIV −	Step 1			Step 2			Step 3		
Parameter	β (SE)	OR	*p*	β (SE)	OR	*p*	β (SE)	OR	*p*
Covariates									
Lifetime AUD	**1.20 (0.48)**	**3.32**	**.012**	**1.07 (0.50)**	**2.92**	**.030**	**1.06 (0.49)**	**2.90**	**.031**
Sensation-seeking T	** − 0.56 (0.25)**	**0.54**	**.011**	** − 0.56 (0.26)**	**0.57**	**.029**	** − 0.56 (0.26)**	**0.57**	**.030**
Independent effects									
MDD				0.68 (0.46)	1.97	.144	0.66 (0.47)	1.93	.165
Pumps				− 0.44 (0.24)	0.64	.070	− 0.39 (0.29)	0.68	.187
Interaction effect									
MDD × pumps							− 0.16 (0.52)	0.85	.758
Model fit									
Pseudo-*R*^2^	0.12		.005	0.17		.003	0.17		.008
Log-likelihood	− 65.36			− 62.72			− 62.67		
Log-likelihood change				2.54		.071	0.05		.757
**Group: HIV + **	**Step 1**			**Step 2**			**Step 3**		
**Parameter**	**β (SE)**	**OR**	***p***	**β (SE)**	**OR**	***p***	**β (SE)**	**OR**	***p***
Covariates									
METH use disorder	**0.97 (0.44)**	**2.63**	**.027**	0.85 (0.46)	2.34	.065	**1.03 (0.48)**	**2.80**	**.033**
AIDS diagnosis	0.78 (0.40)	2.18	.054	0.73 (0.42)	2.07	.084	0.65 (0.43)	1.92	.134
Sensation-seeking T	** − 0.53 (0.24)**	**0.59**	**.027**	** − 0.53 (0.25)**	**0.59**	**.035**	** − 0.55 (0.26)**	**0.58**	**.036**
Independent effects									
MDD				0.35 (0.42)	1.42	.405	− 0.61 (0.58)	0.54	.290
Pumps				− 0.41 (0.22)	0.66	.062	− 0.28 (0.27)	0.75	.283
Pumps^2^				**0.38 (0.17)**	**1.46**	**.026**	0.11 (0.20)	1.12	.575
Interaction effects									
MDD × pumps							− 0.60 (0.59)	0.55	.308
MDD × pumps^2^							**1.20 (0.51)**	**3.32**	**.019**
Model fit									
Pseudo-*R*^2^	0.15		.003	0.22		.002	0.28	< 0.001	
Log-likelihood	− 74.66			− 70.95			− 68.43		
Log-likelihood change				3.71		.059	2.52		.014

Covariates in step 1 were selected using backward selection guided by Akaike’s information criterion. The optimal step 1 model was based on which combination of covariates yielded the lowest overall model AIC value. Logits (β) and odds ratio (OR) estimates for continuous independent variables reflect the effect on NCI for a 1 standard deviation change in the variable (sensation-seeking T: 10-unit change; pumps: 14-unit change). The quadratic pumps term did not reach statistical significance in the HIV- stratum model (*p* = .812) and was therefore removed in order to accurately estimate the linear pumps term. In the HIV − stratum, eight variables were considered as covariates in step 1 because they demonstrated at least a trend-level association (*p* < .10) with MDD (less education, METH use disorder, lifetime non-METH substance use disorder), BART adjusted pumps (younger age) or NCI (male sex, lower estimated premorbid verbal IQ, lower sensation-seeking behaviors, METH use disorder, lifetime alcohol use disorder). For the HIV + stratum, seven variables were considered as covariates because they demonstrated at least a trend-level association (*p* < .10) with BART adjusted pumps (male sex, higher estimated premorbid verbal IQ, higher sensation-seeking behaviors, lifetime non-METH substance use disorder, absence of AIDS) or NCI (older age, lower premorbid estimated verbal IQ, lower sensation-seeking behaviors, METH use disorder, AIDS diagnosis, lower nadir CD4 count). No potential covariates related to MDD status

*AUD* alcohol use disorder, *MDD* major depressive disorder, *METH* methamphetamine

**Fig. 1:**
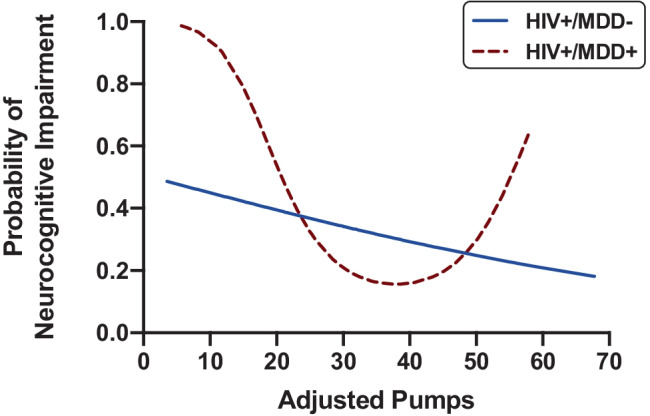
Low and high risk-taking on the BART increase probability of neurocognitive impairment in HIV + /MDD + individuals but not HIV + /MDD −

For the HIV + stratum, the overall fit of the AIC-guided step 1 model was significant (*χ*^2^ = 14.03, Nagelkerke pseudo-*R*^2^ = 0.15, *p* = 0.003). With respect to retained covariates, higher sensation seeking behavior T-scores related to lower odds of NCI and diagnoses of AIDS and METH use disorder related to higher odds of NCI. Step 2 entered MDD status and linear and quadratic adjusted pumps terms as predictors of NCI. The overall model fit of step 2 was marginally improved from step 1 (*χ*^2^ = 21.46, Nagelkerke pseudo-*R*^2^ = 0.22, *p* = 0.002, log-likelihood change *p* = 0.059) and indicated a significant quadratic effect of adjusted pumps (OR = 1.46, *p* = 0.026), but no independent main effect of MDD (OR = 1.42, *p* = 0.405) on odds of NCI. In step 3, the interaction between MDD and the quadratic effect of adjusted pumps significantly related to odds of NCI (OR = 3.32, *p* = 0.019) and resulted in a significant improvement in overall model fit (*χ*^2^ = 28.63, Nagelkerke pseudo-*R*^2^ = 0.28, *p* < 0.001, log-likelihood change *p* = 0.014). In the HIV + /MDD + group, the quadratic effect of adjusted pumps on odds of NCI (OR = 3.72, *p* = 0.005) exhibited a “U-shaped” pattern (Fig. 1) such that the probability of NCI was highest at the lower end of the adjusted pumps range but also elevated at the high end of the adjusted pumps range compared to intermediate levels. Conversely, there were no significant quadratic (*p* = 0.575) or linear effects (*p* = 0.361) of adjusted pumps on NCI in the HIV + /MDD − group.

### BART pumps and neurocognitive domains in HIV + /MDD +

**Fig. 2:**
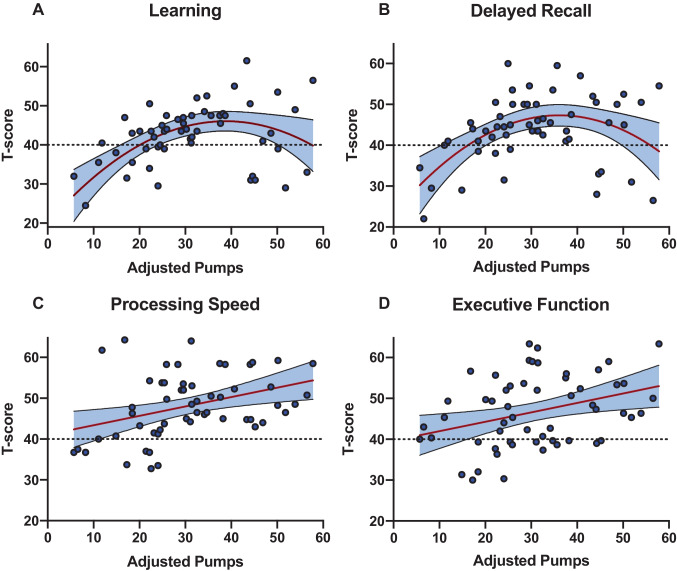
Linear and quadratic effects of risk-taking on domain-specific neurocognition in HIV + /MDD +

To determine which specific neurocognitive domains were driving the observed association between BART adjusted pumps and global NCI in the HIV + /MDD + group, we conducted separate linear regression models with adjusted pumps (linear and quadratic) as the independent variable and neurocognitive domain T-scores as the outcome variables. The combination of linear and quadratic terms for adjusted pumps accounted for a significant amount of variance in learning (linear: beta = 0.19, *p* < 0.001; quadratic: beta =  − 0.02, *p* < 0.001; *R*^2^ = 0.30) and delayed recall T-scores (linear: beta = 0.27, *p* = 0.019; quadratic: beta =  − 0.02, *p* < 0.001; *R*^2^ = 0.24). Similar to the pattern observed for NCI, learning and delayed recall were poorest at the lower end of the adjusted pumps range but also poorer at the high end of the adjusted pumps range compared to intermediate levels (Fig. 2). We did not detect significant quadratic associations for the other domains; however, the linear effect of adjusted pumps explained a significant amount of variance in executive functioning (beta = 0.23, *p* = 0.010; *R*^2^ = 0.12) and processing speed T-scores (beta = 0.23, *p* = 0.005; *R*^2^ = 0.14), with higher adjusted pumps relating to higher T-scores. The quadratic effect of adjusted pumps on learning and delayed recall and the linear effect of adjusted pumps on executive functioning and processing speed remained significant after FDR-correction (*p*s < 0.05). Associations between adjusted pumps and other domains (i.e., verbal fluency, working memory, and motor) did not reach statistical significance.

### BART pumps and HIV transmission risk behaviors

**Fig. 3:**
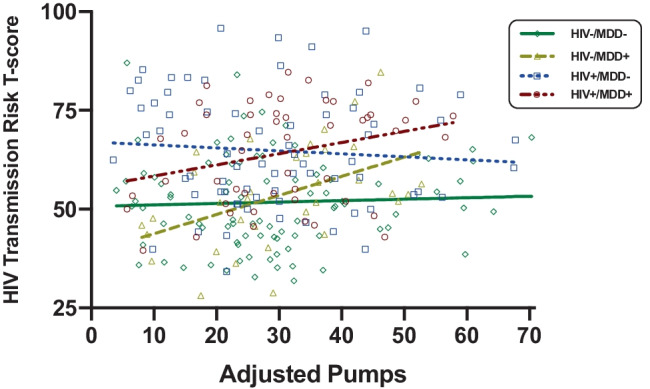
Higher risk-taking on the BART is associated with higher HIV transmission risk behaviors only in MDD + individuals

To examine the ecological relevance of the BART, we examined linear and quadratic associations between BART adjusted pumps and self-reported risky sexual behaviors across the entire study sample and within the four study groups. Higher adjusted pumps was linearly associated with higher HIV transmission risk behaviors T-scores in both MDD + groups (HIV − /MDD + : beta = 0.48, *p* = 0.005, *R*^2^ = 0.23; HIV + /MDD + : beta = 0.28, *p* = 0.046, *R*^2^ = 0.08), but did not relate to HIV transmission risk behaviors T-scores in the full sample (beta = 0.10, *p* = 0.113, *R*^2^ = 0.01) or within either MDD- group (HIV − /MDD − : beta = 0.04, *p* = 0.660, *R*^2^ = 0.00; HIV + /MDD − : beta =  − 0.08, *p* = 0.515, *R*^2^ = 0.01). Quadratic associations between adjusted pumps and HIV transmission risk behaviors T-scores did not reach statistical significance in the full sample or within any of the study groups (*p*s > 0.08). Given the differential pattern of results (Fig. 3), we formally tested for the interaction between MDD and the linear effect of adjusted pumps in the entire sample, controlling for HIV serostatus and METH use disorder. This model indicated a significant interaction effect between MDD and adjusted pumps (beta = 0.38, *p* = 0.002) on HIV transmission risk behaviors in the presence of independent effects of HIV serostatus (beta = 12.34, *p* < 0.001) and METH use disorder (beta = 8.23, *p* < 0.001).

### Secondary analyses: methamphetamine use

Although statistical covariation for METH use did not attenuate the significant associations of BART performance with NCI and HIV transmission risk behaviors, METH use has known adverse effects on neurobehavior and may moderate the relationships between our primary study variables. Thus, a series of post hoc analyses probed the moderating effects of METH use on significant associations established in the primary study analyses. Similar to the lack of HIV/MDD group differences in BART performance, METH use disorder did not significantly relate to BART adjusted pumps in the full sample (METH − mean [SD] = 29.4 [13.6] vs. METH + mean [SD] = 29.9 [14.8], *d* = 0.03, *p* = 0.804) or within each HIV/MDD group (*p*s > 0.426). METH use disorder did not significantly interact with the quadratic effect of BART adjusted pumps on NCI in the HIV + /MDD + group (OR = 1.43, *p* = 0.755), and the quadratic effect of adjusted pumps on NCI in the HIV + /MDD + group persisted in sensitivity analyses stratified by METH − (*n* = 30; OR = 3.73, *p* = 0.037) and METH + (*n* = 26; OR = 4.90, *p* = 0.097). Similarly, METH use disorder did not further moderate the MDD x BART adjusted pumps interaction on HIV transmission risk behaviors T-scores in the full sample (beta = 0.19, *p* = 0.446), and the MDD × BART adjusted pumps interaction persisted in sensitivity analyses stratified by METH − (*n* = 140; beta = 0.29, *p* = 0.073) and METH + (*n* = 102; beta = 0.24, *p* = 0.011).

## Discussion

The present study adds to the literature on risk-taking in HIV by characterizing the concordance of NCI and risk-taking propensity, as indexed by BART adjusted pumps, in a cohort of individuals stratified by HIV serostatus and MDD. Neurocognition was strongest in the HIV − /MDD − group, particularly compared to the HIV + groups, whereas BART adjusted pumps did not significantly differ across the four study groups. Initial observation of the data across the entire study sample suggested that those with NCI exhibited fewer BART adjusted pumps than those without NCI. However, after stratifying by HIV serostatus and examining curvilinear BART effects on NCI, a quadratic pattern emerged in the HIV + group such that rates of NCI were elevated among PWH whose average adjusted pumps fell in the lower end (“risk-averse”) or the higher end (“risk-taking”) of the BART adjusted pumps range compared to intermediate levels. Furthermore, interaction effects indicated that this quadratic pattern of risk-taking propensity and NCI was unique to the dual-risk HIV + /MDD + group and predicted NCI above and beyond comorbid METH use disorder, a history of AIDS, and sensation-seeking traits. Conversely, BART adjusted pumps only exhibited a trend-level linear association with NCI in HIV- individuals that was not moderated by MDD status. Overall, these findings further support a role for affect in modulating HIV-related neurobehavioral functioning and suggest that low and high risk-taking phenotypes are both indicators of neurocognitive dysfunction when lifetime syndromic depression is superimposed upon HIV disease.

Our stepwise regression models indicated a notably large moderation effect of depression on the association between the BART and NCI in PWH. The pseudo-*R*^2^ effect size estimate almost doubled from the step 1 model with only covariates (pseudo-*R*^2^ = 0.15) to the step 3 model that included the MDD interaction with linear and quadratic adjusted BART pumps (pseudo-*R*^2^ = 0.28). Moreover, the quadratic BART effect indicated large alterations to the BART and NCI relationship as a function of BART pumps and 24–30% of the variance in learning and delayed recall was explained by the quadratic BART effect. Neurocognitive domain-specific analyses suggested neurocognitive profiles differed across the spectrum of risk-taking propensity in the HIV + /MDD + group. Individuals with average adjusted pumps on the BART that fell within the lower range had the highest probability of NCI and accordingly exhibited diffuse decrements across the domains of processing speed, executive function, learning, and memory. Strenziok et al. (2011) similarly reported that patients with behavioral variant frontotemporal dementia exhibited fewer BART adjusted pumps compared to controls and that impaired stimulus-reinforcement learning across the task related to greater atrophy in the right lateral orbitofrontal cortex (Strenziok et al. 2011), a region directly implicated in the pathogenesis of depression (Feffer et al. 2018; Yu et al. 2018). HIV + /MDD + individuals with high BART adjusted pumps also exhibited an elevated risk for NCI, yet this risk was driven primarily by poor learning and memory in the context of better processing speed and executive function.

The dissociation between poor learning/memory and intact executive function/processing speed in the high risk-taking HIV + /MDD + group is particularly notable when considering that the BART exhibited a positive, linear association with HIV transmission risk behaviors in this group. This converges with two prior studies that reported better IGT performance related to *greater* risky behaviors (i.e., sexual risk-taking, substance use) in PWH with high levels of emotional distress or sensation seeking personality traits (Golub et al. 2016; Wardle et al. 2010). Several component processes are involved in risky decision-making, including the valuation of potential outcomes, perceived likelihood of outcomes, motivation to pursue positive outcomes, tolerance for negative outcomes, and capacity to learn from past outcomes (Banich and Floresco 2019; Orsini et al. 2019). These data in conjunction with our findings suggest that certain aspects of the cognitive circuitry underlying risky decision-making, such as efficiently processing information to assign values to potential outcomes and subsequently formulating a plan to pursue high-reward albeit risky outcomes, must be intact in order for emotional factors to influence the execution of risky behaviors. In contrast, the poor learning and memory in the HIV + /MDD + group may be less critical for the execution of a risky behavior and may rather reflect an impaired capacity to learn from past negative outcomes, thereby also contributing to risk-taking propensity.

Although performance on behavioral risk tasks may reflect acquired neurocognitive deficits that underpin the component processes of decision-making, the somatic marker hypothesis describes the strong influence of affective states on risky decision-making (Bechara et al. 2000; Lerner et al. 2015). This hypothesis postulates that when confronted with a decision, individuals receive bioregulatory signals (i.e., somatic markers) rich in affective information regarding possible response outcomes (Bechara et al. 2000; Damasio 1996). Under this somatic marker framework, suboptimal decision-making may manifest from the failure to integrate these somatic markers with the cognitive components of decision-making, particularly in the setting of monoaminergic dysfunction (Bechara et al. 2000; Rogers et al. 1999). For example, depressed individuals may ineffectively process affective signals during decision-making due to altered reward and/or punishment sensitivity arising from disrupted cortico-striatal reward signaling (Eshel and Roiser 2010; Husain and Roiser 2018; Must et al. 2006). Importantly, this may help explain the heterogeneity in risk-taking and neurocognitive performance observed in our historically-depressed group of PWH, as altered reward processing may generate affective signals that contribute to both risk averse (Hevey et al. 2017; Smoski et al. 2008) and risk-taking behaviors (Whittle et al. 2015), particularly in PWH (Cook et al. 2015).

The relationship between BART performance and NCI in individuals without HIV was weaker than our observations in PWH and was not moderated by lifetime MDD status. From a statistical perspective, the specificity of our findings to PWH may be partially explained by higher rates of NCI in HIV + /MDD − and HIV + /MDD + compared to the HIV − /MDD − group, which comprised 72% of the HIV − sample. Furthermore, the HIV − /MDD + reported similar levels of current depressive symptoms to the HIV + /MDD- group and substantially milder symptoms compared to the HIV + /MDD + group. The higher levels of current neurocognitive and affective dysfunction seen in the HIV + /MDD + sample likely enhanced power to detect MDD-dependent associations between the BART and neurocognition, which would have been less likely had groups been classified based on current MDD criteria given the small sample size. Nevertheless, BART adjusted pumps explained 23% of the variance in risky sexual behaviors in the HIV-/MDD + group, underscoring the convergent validity of the BART with real-world risk behaviors in individuals with histories of clinical depression.

Although partially explained by the above mentioned findings, the lack of differences across groups on BART performance may be explained by additional factors, and is worth discussion, particularly given inconsistencies in the literature. For example, some studies have reported that PWH, particularly those with elevated affective distress, exhibit greater risk-taking across several behavioral risk tasks (Hardy et al. 2006; Martin et al. 2016; Paydary et al. 2016; Thames et al. 2012). Others have failed to identify main effects of HIV serostatus on risky decision-making, particularly the IGT, yet have detected HIV-related influences on risk-taking that are conditional on neurocognitive and affective factors (Gonzalez et al. 2005; Iudicello et al. 2013). This is consistent with our observations and highlights the importance of understanding the affective and neurocognitive conditions that contextualize BART performance, as examination of BART performance in isolation did not adequately discriminate HIV-related and MDD-related neurobehavioral dysfunction. There are other potential explanations for the lack of group differences across BART performance. Specific to our study, our comparison groups (e.g., HIV − , MDD −) were drawn from the same parent study as our groups of interest (TMARC), which overall had high proportions of substance use (e.g., METH, alcohol) use disorders and elevated behavioral characteristics (e.g., impulsivity, sensation seeking) that have been strongly associated with risk taking propensity on the BART (Kohno et al. 2014; Lejuez et al. 2002). The lack of group differences may also be attributed to the BART outcome measure used (e.g., average adjusted number of pumps), which while extensively used in the literature, may not fully capture the intricacies of risk-taking propensity in these populations. Future research exploring other metrics, such as those aimed at elucidating the profile of performance on the BART (e.g., inter-trial variability or differences in behavior following balloon explosions versus rewards; Canning et al. 2021), could provide valuable insight into risky decision-making in these populations, as would the development of computational models that tap into multiple facets of risk-taking propensity (Park et al. 2021; Wallsten et al. 2005).

Growing evidence indicates that a subset of clinically depressed patients display an enhanced inflammatory state that is linked to monoaminergic dysregulation in corticostriatal circuits underpinning motivation and reward (Felger et al. 2016; Felger and Miller 2012; Haroon et al. 2018). Consistent with this immunophenotype of depression, we have recently characterized associations between HIV-associated neuroinflammation, depressive symptoms, and CSF dopaminergic deficits (Ellis et al. 2020; Saloner et al. 2020a). Moreover, chronic depression predicts steeper declines in executive function and recall and moderates the acute effects of systemic inflammation on psychomotor and cognitive slowing in PWH (Paolillo et al. 2020; Saloner et al. 2020a). Risk-taking profiles may show high concordance with neurocognitive dysfunction in depressed PWH given that HIV-associated neuroinflammation preferentially targets these frontostriatal circuits that support higher-order neurocognitive functions, emotional regulation, and reward processing (Soontornniyomkij et al. 2016; Woods et al. 2009). A recent study in stimulant-using PWH identified an association between higher BART scores and greater tryptophan degradation (Lee et al. 2020), a marker of immune activation and serotonin deficiency that is implicated in HIV-related depression (Gostner et al. 2015). At the neurocircuit level, HIV disease has been associated with reduced resting-state frontostriatal connectivity, which in turn correlated with higher odds of NCI (Ipser et al. 2015). PWH also exhibit greater activation of the prefrontal cortex during a monetary decision-making task, suggestive of decreased neural efficiency, and this compensatory neural activation of the prefrontal cortex is related to higher BART scores and lower nadir CD4 counts (Meade et al. 2016).

This study is among the first to model linear and quadratic patterns of risk-taking propensity, measured with the BART, as it relates to neurocognition and real-world risk-taking in a well-characterized cohort of individuals stratified by HIV serostatus and MDD. It is worth noting several limitations to the present study. Due to the overarching parent study (TMARC) aims (Saloner et al. 2020b; Soontornniyomkij et al. 2016), a significant proportion of participants had METH use disorder diagnoses, including the HIV- and MDD- samples. METH use is associated with risk-taking behaviors (Gonzalez et al. 2007) and increases risk for NCI in PWH (Carey et al. 2004; Rippeth et al. 2004). Consistently, METH use was associated with NCI in our study, but only in the HIV + group. However, our main study findings, namely, the quadratic BART association with NCI in the HIV + /MDD + group, persisted while controlling for METH use as a covariate. These findings were further strengthened by sensitivity analyses demonstrating that the quadratic BART association with NCI in the HIV + /MDD + group persisted in both METH − and METH + individuals. To provide further support for our findings in light of the high prevalence of METH use in our sample, we conducted post hoc analyses to probe the potential influence of METH use on risk-taking propensity and potential interactive effects with significant associations established in the primary study analyses. Similar to the lack of HIV/MDD group differences in BART performance, METH use disorder did not significantly relate to BART adjusted pumps in the full sample or within each HIV/MDD group. Moreover, METH use disorder did not significantly interact with the quadratic effect of BART adjusted pumps on NCI in the HIV + /MDD + group. Lastly, neither duration (i.e., lifetime total days of METH use) and amount (lifetime total grams consumed) related to probability of NCI or BART adjusted pumps, regardless of HIV or MDD status (data not shown). While METH use diagnosis and characteristics did not appear to moderate our primary findings, it is nonetheless essential to consider in future studies examining risk taking propensity or profiles of risk behavior, particularly in HIV and other populations in which use is highly prevalent. Other factors such as personality traits associated with METH use (e.g., reward sensitivity) may also play a role in risk propensity (e.g., White et al. 2007) and should be carefully considered in future research.

Second, our data importantly integrates an experimental cognitive task (i.e., BART) standard clinical neuropsychological tests, and ecologically relevant risky sexual behaviors, yet these neurobehavioral data do not directly measure the frontal systems neurocircuitry implicated in HIV and depression. Future studies that incorporate biological units of analysis, such as structural/functional neuroimaging and inflammatory biomarkers, would allow us to formally examine the putative neurobiological mechanisms underpinning risk-taking behavior in this population. Next, the cross-sectional design of our study does not allow us to confirm the directionality or stability of our findings. We intentionally entered BART performance as a predictor of neurocognition in order to examine quadratic patterns of risk-taking propensity; however, it is also reasonable to conceptualize neurocognition as a determinant of risky decision-making and therefore our independent and dependent variables are not discussed in terms of causality. Our study population of PWH was also predominantly men, consistent with the demographics of PWH in San Diego, with a high prevalence of comorbid substance use disorders. Prior studies have described sex differences in IGT performance and NCI in PWH (Martin et al. 2016; Rubin et al. 2019; Sundermann et al. 2018) as well as sex influences on BART performance in HIV-seronegative adults (Lejuez et al. 2002; Lighthall et al. 2009; White et al. 2007), and future work should therefore aim to elucidate sex differences in the neurocognitive correlates of BART performance in PWH. Last, our overall study sample likely reflects a group high in characteristics associated with risk-taking, higher baseline levels of risk-taking, NCI, and emotional dysregulation than a sample without a history of addictive behaviors. The common co-occurrence of substance use disorders in PWH with and without MDD enhances the ecological validity of our findings, however future studies focused on MDD in people without significant substance disorders should help to validate the specificity of the relationships that we report. In addition to risky sexual behaviors, future analyses should examine the moderating influence of lifetime and current MDD on the relationship between BART performance and other facets of real world risk-taking such as substance use behaviors in PWH.

Our findings challenge prior studies that have assumed a linear relationship between risk-taking propensity and neurocognition, particularly in groups with underlying frontostriatal dysfunction. Moreover, these findings advocate for neurobehavioral screenings that incorporate both affective and neurocognitive measures in PWH. Given the large influence of depression on the relationship between risk-taking and neurocognition, clinicians may consider further screening for risky behaviors in PWH who present with comorbid depression histories and intact executive functioning/processing speed but impaired learning/memory. Interventions aimed at ameliorating depressive symptoms and/or enhancing sensitivity to the adverse consequences of risk-taking may help reduce HIV transmission risk in these individuals. Conversely, the absence of risky decision-making in depressed PWH may not purely reflect low risk-taking propensity, but may also reflect overlapping neurocognitive and depressive symptoms (e.g., executive dysfunction and apathy) that contribute to impaired decision-making. Thus, diffusely impaired and “deactivated” individuals should also be monitored in the event that they experience “activations” in aspects of their neurocognitive and affective profile that facilitate the execution of risky behaviors.
